# Changes in clients' perceptions of family planning quality of care in Kaduna and Lagos States, Nigeria: A mixed methods study

**DOI:** 10.3389/fgwh.2022.1034966

**Published:** 2022-11-22

**Authors:** Toyin O. Akomolafe, Funmilola M. OlaOlorun, Emeka Okafor, Sikiru Baruwa, Kayode Afolabi, Aparna Jain

**Affiliations:** ^1^ Research Impact, Population Council, Abuja, Nigeria; ^2^ Department of Community Medicine, College of Medicine, University of Ibadan, Ibadan, Nigeria; ^3^ Programs Department, Society for Family Health, Abuja, Nigeria; ^4^ Reproductive Health, Federal Ministry of Health, Abuja, Nigeria; ^5^ Reproductive Health Program, Population Council, Washington DC, United States

**Keywords:** family planning, quality of care, private sector, community pharmacist, drug shop owners

## Abstract

Community Pharmacists (CPs) and Patent and Proprietary Medicine Vendors (PPMVs) are crucial to improving access to family planning (FP) services in Nigeria. Although the private sector is preferred for convenience, availability of commodity, privacy, and timeliness, less well known is the quality of care received by clients who obtain FP services from CPs and PPMVs. This paper seeks to explore the use of validated quality of care measures for programming in Kaduna and Lagos States and to assess how these measures worked in capturing changes in quality of care using client exit interviews implemented at two time points. Using validated measures of quality of care, 598 and 236 exit interviews in rounds 1 and 2 were conducted with FP clients aged 18–49 years old. The quality of care domains were assessed using 22 questions. A weighted additive quality score was created, and scores were grouped into three: low, medium, and high quality. Changes in quality of care received were examined using *χ*^2^ test. A subset of 53 clients were selected for in-depth interviews. Deductive and inductive approaches were used for coding, and data analysis was thematic. In Lagos, we observed increases in 16 out of 22 items while in Kaduna increases were only observed in 8 items. For instance, increases were observed in the proportion of women who experienced visual privacy between rounds 1 and 2 in Lagos (74%–89%) and Kaduna (66%–82%). The quality of care received by clients changed over time. Women who reported high quality care in Lagos increased from 42% to 63%, whereas women who reported high quality care in Kaduna decreased from 35% to 21%. In both states, in-depth interviews revealed that women felt they were treated respectfully, that their sessions with providers were visually private, that they could ask questions, and that they were asked about their preferred method. This study demonstrates that clients received high quality of care services from providers (CPs and PPMVs) especially in Lagos, and such services can be improved over time. Continuous support may be required to maintain and prevent reduction in quality of FP counseling and services, particularly in Kaduna.

## Introduction

The role of the private sector in improving access to quality family planning (FP) services and reducing inequities in access and use is becoming more prominent. In Nigeria, more than half (54%) of private sector outlets stock modern contraceptives, though general retailers only stock condoms (1). Patent and Proprietary Medicine Vendors (PPMVs) or drug shop owners, comprise 71% of the total market for modern contraceptives in Nigeria (1). In 2018, forty-one percent of modern contraceptive users in Nigeria obtained family planning services from the private sector, which is the main source of many reversible methods (2). Over one-third (34%) of modern contraceptive users received their last method from a PPMV or Community Pharmacist (CP) (2). PPMVs operate legally in Nigeria and are defined as persons without formal training in Pharmacy who sell orthodox pharmaceutical products on retail basis for profit (3). PPMVs do not require more than secondary education to operate and current regulations only permit them to sell a limited number of pre-packaged, over-the-counter medicines and medical products, but prohibits them from selling prescription medications (e.g., antibiotics) or conducting invasive medical procedures (4, 5). Recent studies show that one out of four PPMVs have health qualification and previous experience working in a health facility (6). CPs have a formal degree in pharmacy, mostly operate in urban areas, and are licensed by the Pharmacists Council of Nigeria (PCN) to sell prescription drugs (4). PPMVs are located close to communities and are often the first source of care for hygiene and FP products and treatment of childhood illnesses (4). Despite the benefit of scale and patronage, the quality of health services provided by PPMVs is low (7).

Studies have shown that higher levels of quality of care received are associated with modern contraceptive uptake and continuation (8–11). In Kenya, a strong association was reported between contraceptive use and aspects of quality of service; information provided during method selection, effective method use and potential side effects, and being treated well during service provision, particularly among young and less educated women (8). Using five aspects of quality of care (needs assessed, information received, method choice, interpersonal relationships, and continuity of care), a panel study in the Philippines showed that increased quality of care was associated with increased continued use of modern contraceptives (11). Method information index (MII), a key component of quality of FP care received, was associated with continued contraceptive use (12–14). For example, high MII scores was associated with low implant discontinuation rates in Kinshasa (12) and risk of discontinuation decreased with higher MII score among women in Pakistan and Uganda (13).

Two widely referenced frameworks that have laid the foundation for defining quality of care are the Donabedian and Bruce frameworks. Donabedian's framework identified three components for measurement of quality of care: (i) structure (physical infrastructure, equipment, commodities and organizational capacity), (ii) process (interactions between providers and patients, and method of service provision), and (iii) outcomes (effect of care on health status of patients) (15). Bruce's quality of care framework developed specifically for FP, consists of six elements: (i) technical competency of providers, (ii) combination of appropriate constellation of services, (iii) information given to users, (iv) choice of methods, (v) interpersonal relations, and (vi) follow-up or continuity mechanisms (16). As rights-based family planning programming has evolved, it has been proposed that the six elements of the Bruce framework can be regrouped into two levels; structure and service-giving process (quality of care), and modified to include: availability of trained providers with attention to infection prevention practices, solicitation of information from clients, option of switching of methods in continuity of care, confidentiality and privacy, provision of intended standard of care, and information exchange between providers and clients (17, 18). Four domains were proposed to measure the service-giving process level of the modified Bruce-Jain framework: respectful care, method selection, effective use of method selected, and continuity of contraceptive use and care (16, 17, 19, 20).

The need to measure quality of care in routine monitoring is an important component of quality improvement. This paper seeks to explore the use of validated quality of care measures for programming and to assess how these measures work in capturing changes in quality of care using client exit interviews implemented at two time points. Also, this paper seeks to provide in-depth insights on quality of care received by clients who seeks FP services from CPs and PPMVs.

## Materials and methods

### Intervention

The IntegratE project aimed to increase access to contraceptive methods in underserved areas of Kaduna and Lagos states in Nigeria through the involvement of private sector providers: CPs and PPMVs. The project supported by the Bill & Melinda Gates Foundation and MSD for Mothers, worked alongside the government and other stakeholders to provide standardized training in family planning counselling and method options to CPs and PPMVs. The project built upon an ongoing effort to expand access to modern contraception, through increasing the number of service delivery points providing FP services and inclusion of PPMVs in task shifting policy implementation, which is essential to achieving Nigeria's commitment to modern contraceptive prevalence rate (mCPR) of 27% among all women by 2024 (21). Working in collaboration with the regulatory body, Pharmacists Council of Nigeria (PCN), the Project piloted a three-tier accreditation system for PPMVs based on their healthcare qualifications. PPMVs were grouped into three tiers based on their health qualifications and trained on counseling techniques, family planning methods: use, efficacy of methods and side effects, depending on their cadre. The three tiers of PPMVs were ranked from Tier 1 (lowest) to Tier 3 (highest) in line with their health qualifications. CPs are outside of the accreditation system and received similar trainings to PPMVs with health qualifications. Except for non-health trained PPMVs which are grouped as Tier 1, all other PPMVs and CPs received training on injectables and implants. Between January and June 2019, IntegratE project trained 894 CPs and PPMVs from Kaduna and Lagos states. A detailed description of IntegratE Project, the three-tier accreditation system, description of tiered PPMVs and type of training received can be found elsewhere (22).

### Study settings

The first phase of the IntegratE project was implemented in Lagos and Kaduna states, and both were selected as study sites. Lagos State has the highest modern contraceptive rate in Nigeria (29%), a fertility rate of 3.4, and 17% of married women who wanted to delay or stop childbearing are not using family planning (2). Compared to the national average and other regions, women in the Southwest region where Lagos State is located have a high desire to limit childbearing (37%) and this desire was 88% among women with six or more children (2). In Kaduna, 14% of married women use a modern contraceptive method, fertility rate was 5.9, while 12% had an unmet need for FP. In the Northwest, women have a low desire to limit childbearing (16%), with 39% of women with six or more children (2). PPMVs, the foremost source of modern contraceptives in the private sector, had an average of 39 shops per 100,000 population in both states: 46 shops per 100,000 population in Kaduna State and 32 per 100,000 in Lagos State (6).

### Study design

A mixed methods study was designed to assess the quality of care received by FP clients who sought services from IntegratE trained CPs and PPMVs in Kaduna and Lagos states. The study is part of implementation science. The data for this paper utilized two rounds of client exit interviews conducted between June and November 2019 (round 1) and between November 2020 and February 2021 (round 2). Both rounds of data collection included a fresh sample of exiting clients. Quantitative interviews were conducted with clients within two weeks of receiving FP services from trained CPs and PPMVs in both rounds. The first round of exit interviews was conducted shortly after providers were trained. The second-round of exit interviews were conducted after providers had settled into their new role of providing FP services especially long-acting reversible contraceptive (LARC) methods. Qualitative interviews were conducted with a subset of women who completed the quantitative interviews from December 2020 to July 2021. The qualitative data were not collected with the intention of measuring change over time but to gain insights into clients' perception of quality.

### Data collection

Quantitative interviews were conducted by phone. Prior to each round of data collection, six interviewers were trained for two to three days on study objectives, tools and obtaining informed consent. Open Data Kit (ODK) Collect, a mobile data collection application was used, and the data entry form was designed to include automatic skip patterns and restrictions for completeness. FP clients were recruited through CPs and PPMVs who requested permission to share their contact details with the research team. Women aged 18 to 49 years old were contacted by phone by trained interviewers, provided with information about the study and interviewed in English, Hausa or Yoruba, depending on their preference, after obtaining informed consent. Information was obtained regarding participants' background characteristics, type of method received, experiences receiving FP services from the CP/PPMV that they saw, and the quality of care received. Responses were entered using ODK app in an electronic device. Completed questionnaires were uploaded into the ODK online server daily, which the study coordinator reviewed for quality.

In-depth interviews were conducted by three research assistants with previous experience in qualitative research. They were trained for two days on the study objectives, study tools, interview techniques, and the peculiarities of conducting interviews by phone where non-verbal cues would be more difficult to capture. A subset of women who participated in the survey were identified and purposively selected based on their willingness to participate. An in-depth interview guide was used to obtain information on the experiences of women who received FP services from trained CPs and PPMVs. Women were asked about the quality of care received, personal interactions with the provider, satisfaction with their selected method, partner's support, and economic impact of the COVID-19 pandemic. All interviews were conducted by phone in English, Yoruba, Hausa, or Pidgin English. At the start of each interview, informed consent was received from each respondent, and interviews were audio recorded. Verbatim transcriptions were done, including translations where indicated. All transcripts were read by the study supervisor and research assistants were provided with feedback to improve their interview skills during the period of data collection.

The exit interview questionnaire and in-depth interview guide used were developed by the IntegratE project research team, to answer the study objectives. The questions for the survey were adopted from previous tools with content validity established. The tools were shared and reviewed internally, and feedback was incorporated. To ensure face validity, both tools were shared with content experts for further refinement, with items revised, removed or added based on feedback received. The tools were pretested on a sample of clients with characteristics similar to those of the proposed study population, and further revisions made. Due to the advent of COVID-19, data were collected through phone interviews.

Ethical approval for the study was received from Population Council Institutional Review Board and the National Health Research Ethics Committee (NHREC) of the Federal Ministry of Health.

### Measures

#### Quality of care measures

Quality of care received from CPs and PPMVs was measured using the four domains proposed for measurement of the service-giving process (quality of care) level of the Bruce-Jain framework. The domains consist of 22 items: respectful care (6 items), method selection (7 items), effective use of method selected (5 items), and continuity of contraceptive use and care (4 items) (19, 20). Respectful care focuses on interpersonal relations between provider and client and consists of treatment of clients with dignity and respect, audiovisual privacy, and confidentiality of information shared. Method selection, effective use of method selected, continuity of contraceptive use and care domains reflect information exchange between provider and client. Method selection involves solicitation of information from clients on previous FP experience, preferred FP method, desire for another child, preferred timing of next child, providing information on other FP methods, and methods that protect against STIs. Effective use of the method domain involves informing clients about how to use chosen method, possible side effects and warning signs, and how to manage them. Continuity of contraceptive use and care involves informing about timing of follow up visit, other sources of FP supply, and possibility of switching methods (18, 19). Validation of the 22 items scale was conducted in a longitudinal study of FP clients in India which showed that those who received higher levels of quality were more likely to continue modern contraceptive use 3 months later (20).

Each item was assigned a “1” for quality received and “0” if quality was not received. A weighted additive quality score was calculated for each woman, with each domain assigned equal weight. The average score for each domain was calculated by adding all items in a domain and dividing the sum by the number of items in that domain. The domain averages were multiplied by 100 and divided by the total number of domains to get a quality score, which ranges from 0 to 100 (20, 23). A three-category quality score was constructed from the responses to the 22 quality items for each woman. The quality score was grouped into three categories: low, medium, and high. The low-quality score was zero to mean score minus half of standard deviation and the high-quality score was greater than or equal to mean score plus half of standard deviation (20, 23).

### Covariates

Demographic characteristics of clients include age, marital status, education level, number of living children, and household wealth index. Age of clients was grouped into three categories: 15–24 years, 25–34 years, and 35 and above. Marital status was categorized into three; single, married/in-union, and widowed/divorced. Education level of clients was grouped into four categories: no formal education, primary, secondary, and two plus years post-secondary. The number of living children was grouped into none, one, two, three, and four or more children. Household wealth index was measured using a composite score calculated using principal components analysis of household asset items and was categorized into lowest, middle and highest.

### Data analysis

Descriptive statistics were used to examine the characteristics of the respondents between the two rounds. Bivariate analysis was employed to assess the changes in each quality of care measure in addition to the quality scale by data collected round, and *χ*^2^ was used to test for significance. Analysis was disaggregated by state. Quantitative analysis was done using STATA 16.1 software.

Qualitative data were analyzed for perception of clients. Transcripts were read through several times and data segments coded. Codebook development was iterative, and both deductive and inductive approaches were employed for coding. Using the Bruce-Jain framework for the study, relevant codes were grouped into themes matching the four domains of the quantitative analysis. Coding and qualitative analysis were done using Atlas.ti 8.4.5 software, and representative quotes were identified for each theme.

Quantitative and qualitative findings were organized by the four quality domains and compared for similarities and differences.

## Results

### Respondent characteristics

Table 1 shows the demographic characteristics of women interviewed in client exit interview by state for both rounds. A total of 834 women had visited a CP or PPMV for FP services in the 2 weeks prior to the interview; 371 and 463 in Kaduna and Lagos states, respectively. The majority of women from Kaduna state were interviewed in the first round of exit interviews (80%). Women from Kaduna state were younger and had less education than women from Lagos state. Similar proportions were seen in marital status, number of living children, and household wealth across both states.

**Table 1:** Respondent characteristics by state, survey.

	Kaduna (*n* = 371)	Lagos (*n* = 463)	Total (*N* = 834)
Age			
18–24	18.9	8.4	13.1
25–34	52.8	46.0	49.0
35+	28.3	45.6	37.9
Marital Status^a^			
Single	5.7	4.1	4.8
Married/In-union	91.9	94.6	93.4
Widowed/divorced	2.2	1.3	1.7
Number of living children			
None	4.1	3.2	3.6
One	10.2	8.4	9.2
Two	23.2	18.2	20.4
Three	24.0	31.1	27.9
Four or more	38.5	39.1	38.9
Highest level of education achieved^a^			
No formal education	8.4	3.0	5.4
Primary	21.6	16.6	18.8
Secondary	48.5	39.7	43.7
2 + years post-secondary	20.8	38.4	30.6
Household Wealth Index^a^			
Lowest	35.9	31.3	33.3
Middle	33.7	34.3	34.1
Highest	30.2	34.3	32.5
Period Interviewed			
1st Round	80.3	64.8	71.7
2nd Round	19.7	35.2	28.3

^a^ Percentages do not total to 100% due to missing observations.

The in-depth interviews consisted of 53 women, with majority aged 25 years or more. The demographic characteristics of women who participated in the qualitative interviews are shown in Table 2.

**Table 2:** Demographic characteristics of participants in qualitative interviews.

Characteristic	Kaduna (*n*)	Lagos (*n*)
Age	4	2
<25 years	23	24
≥25 years		
Highest level of education		
Primary or less	1	4
Secondary	16	11
Post-secondary	10	11

### Quality of care received

**Figure 1:**
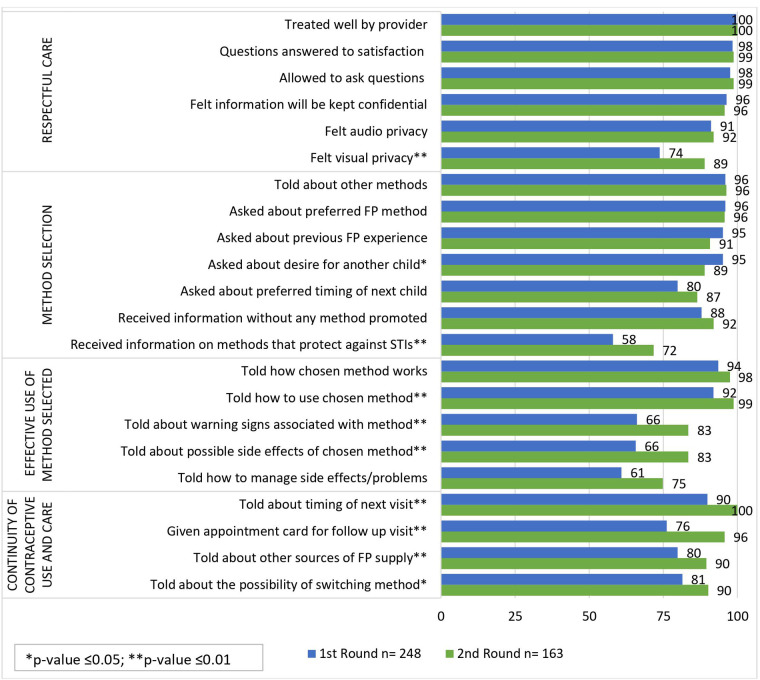
Percentage of women reporting quality of care received from CPs and PPMVs in Lagos by domain and round (*n* = 411).

**Figure 2:**
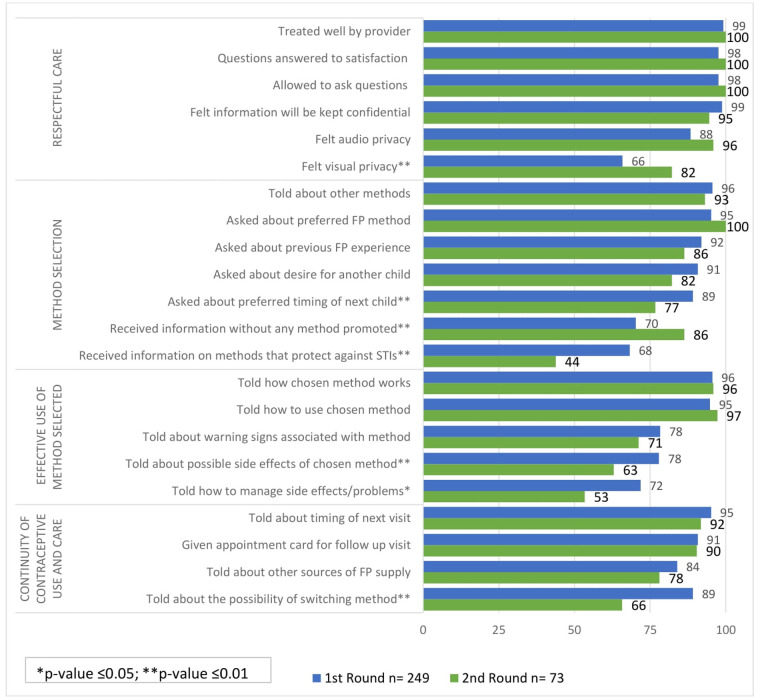
Percentage of women reporting quality of care received from CPs and PPMVs in Kaduna by domain and round (*n* = 322).

Figures 1, 2 show the quality of care received by women who sought services from CPs and PPMVs in Lagos and Kaduna states, respectively. The items are presented by quality of care domain and by data collection round.

#### Respectful care

Most women in both Kaduna and Lagos states reported receiving five out of the six quality items in the respectful care domain including being treated well by the provider, their questions were answered to their satisfaction, they were allowed to ask questions, felt information will be kept confidential, and felt consultation could not be heard by any other clients. Increases across these five items were generally observed between round 1 and round 2 in both states.

The qualitative transcripts indicated that all women interviewed reported being treated well by the CP or PPMV they visited. For those who had questions, women said providers listened to them and answered all their questions satisfactorily. Several women indicated that they did not ask questions during the consultation or did not have questions they wanted to ask. Furthermore, most women felt that their information would be kept confidential, as explained by a woman in the following quote:

“*Honestly, even me, I was able to direct some of my co-wives in the family. But you know things are done confidentially. But some of them went there and they told me they enjoyed her services. I was able to direct two friends of mine and some co-wives in the family, but I can’t say that this [method] is what they choose. Do you understand*?” (27-year-old married implant user; 3 children; secondary education; Kaduna state)

A significantly higher proportion of women in both states reported experiencing visual privacy while receiving care from CPs/PPMVs in round 2 compared to round 1. In Lagos, 89% of women felt visual privacy in round 2 compared to 74% in round 1 (Figure 1). Similarly, in Kaduna, the proportion of women who felt visual privacy in round 2 (82%) was significantly higher compared to round 1 (66%) (Figure 2). The findings from the qualitative interviews corroborated the widespread experience of visual privacy in both states as evidenced by the following quotes:

 “*And she now took me into an office, maybe a small room, where she attended to me privately.”* (43year-old; tertiary education; 4 children; implant user; Lagos state)

 “*Truthfully, there was privacy, it was just me and her alone. Even if people are in the shop, she has an inner room where she will attend to you.”* (28-year-old; married implant user with 3 children and secondary education; Kaduna state)

Despite clients' affirmation of both visual and auditory privacy, narrated experiences showed that auditory privacy may have been compromised. Most women felt their interaction with the provider was private because they entered an inner room to discuss, even when the outer area was full of people. No one specifically elaborated on the possibility that someone on the other side of the divider or wall could have heard what they were discussing with the provider. During these interactions, the provider's apprentice, or another provider may have also been present as illustrated by the following quote:

*“There was privacy at the pharmacy. The day I went, I was the only one in her office that they attended to, there was privacy, no one was there except for the auxiliary nurse working with her.”* (43year-old; tertiary education; 4 children; implant user; Lagos state)

#### Method selection

Method selection refers to the exchange of information between a provider and a client that enables a client to select an appropriate method. Most women in Lagos and Kaduna states reported they were told about other FP methods and that the provider asked them about their preferred FP methods. This was consistent is both data collection rounds.

Qualitative data confirmed that providers offered information about a range of FP methods, although what was shared differed for new and returning clients. While their recollection was not complete, clients who were new users generally reported that they were told about other methods apart from the one they received. Clients who were returning users did not seem to expect to receive much information from the provider, when asked if counselled about other family planning methods, likely since they were coming for resupply of a previously selected method. When asked if she received information about other FP methods, one woman responded as follows:

*“No because I didn’t go as a newcomer. I just told her what I want, and she asked me why and I told her that [I] have used it before.”* (42-year-old; married implant user with 4 children and tertiary education; Lagos state)

The qualitative interviews revealed that clients who reported being counseled on a variety of FP methods also mentioned that they were asked about their preferred method among the methods they had just heard about. While some had made up their mind about which method they wanted, even before seeking services from the provider, others made their decision based on information received from the provider. Yet others reported making a decision jointly with their partners as depicted in the following quote:

"*Yes, my decision, I was…you know, my kids were two—two years interval and I want to kind of give them gap with the last one with me and my husband said we should go ahead for it that we should hold on for kids for now. So, I called him, “should I do the one of five years or three years” and he said we should look for the one of three years first, to know how it will be, so I went for the three years own*.” (32-year-old; married implant user with 2 children and tertiary education; Lagos state)

There are two questions related to fertility intentions that can assist providers in recommending an appropriate FP method—whether women were asked about desire for a child and asked about the preferred timing of the child. In Lagos state, most women interviewed at round 1 and round 2 reported being asked about these two questions. However, there was a slight decrease in the proportion of women who said they were asked about their desire for another child, from 95% in round 1% to 89% in round 2 (*p*-value ≤0.05; Figure 1). In Kaduna state, fewer women also reported to have been asked about the desire for another child at round 2 (91%) compared to round 1 (82%) but this was not a significant difference (Figure 2). Significantly fewer women in Kaduna said they were asked about the timing of their next child at round 2 compared to round 1 (77% vs. 89%, respectively, *p*-value ≤0.01).

In both states, there was an increase in the proportion of women who reported that they received information about FP without any method being promoted in round 2 compared to round 1. There was a significant increase observed only in Kaduna state where the increase was from 70% in round 1% to 86% in round 2 (*p*-value ≤0.01).

In Lagos State, women reported receiving information on methods that protect against sexually transmitted infections (STIs) more in round 2 (72%) compared to round 1 (58%, *p*-value ≤0.01) (Figure 1). Qualitative data, however, showed that some women in Lagos remembered being told about how to protect themselves against STIs but were unable to give details of the conversation while others said they did not receive this information.

*“Yes, they said it (*Sexually Transmitted Infections*), but I may not have listened to all that they discussed with me or even remembered most of the things we discussed”.* (45-year-old; no formal education; 3 children; implant user; Lagos state)

In contrast, women who reported receiving information on methods that protect against STIs in Kaduna reduced significantly from 68% in round 1% to 44% in round 2 (*p*-value ≤0.01).

#### Effective use of method selected

Five questions were explored to assess whether information was provided by the provider to the client for effective use of the method she selected. In Lagos, increases across all five of these questions were observed from round 1 to round 2 (Figure 1). Significant increases were observed from round 1 to round 2 for three of the five questions: told how to use the chosen method, told about warning signs associated with the method, and told about possible side effects of the chosen method.

Qualitative interviews in Lagos state confirm that women were told about warning signs and side effects as indicated in the following two quotes:

*“Yes, they told us that our period (menses) may stop. That it may come 2–3 times in a month. They said we shouldn’t be afraid about the irregular menstrual flow or be afraid that the drug is not effective or that we are pregnant. Their assurance about the method gave me peace of mind but I am still thinking of going back to the premise that since November, the 11th month, I haven’t seen my menstruation. If I go to the premise and they tell me that there is no problem, I will not have been worried. I haven’t seen any sign of pregnancy at the moment and it has not done anything bad to me, the only thing is that I haven’t seen my menses.”* (45-year-old; no formal education; 3 children; implant user; Lagos state)

* “Ah, eh, I was told that some people might be having, eh, stomach, big stomach, might be getting fat, some might be getting lean, is that, is that some might be having slight headache.”* (43-years; tertiary education; 4 children; implant user; Lagos state)

In Kaduna state, a reduction in women who reported receiving three of the five pieces of information was observed from round 1 to round 2. Fewer women were told about the possible side effects of their chosen method in round 2 (63%) compared to round 1 (78%) and fewer women were told how to manage side effects in round 2 (53%, Figure 2).

#### Continuity of contraceptive use and care

Four quality items in the continuity of contraceptive use and care domain were assessed at rounds 1 and 2. In Lagos, significant increases were observed for all four items at round 2 where more women were told about the timing of their next visit, received an appointment card, given information about other sources for resupply and told about the possibility of switching methods. In Kaduna, however, the proportions for two items remained relatively the same—told about timing of next visit and given an appointment card—and reduced at round 2 for told about other sources of FP resupply and told about the possibility of switching. The latter measure reduced significantly from 89% at round 1% to 66% at round 2 (*p*-value <0.01).

Qualitative data suggested inconsistency in information sharing about continuity of care. Overall, women were told about when to return for their next visit, and a few noted that they were given appointment cards for their follow up visit. Some women were told about other sources of FP method supply, especially women who were using implants and IUDs and would require removal, with or without a fresh insertion. Very few women reported being told about the possibility of switching to another method, in the event that they were dissatisfied with the method they were currently using.

Apart from appointment dates for women using injectables, and information on where and when to remove implants and IUDs, many women received very little parting instructions from the provider. Some women were given parting instructions on abstinence or use of back-up method of contraception to protect against pregnancy pending the steady release of hormone from the implants. Furthermore, some women were told to keep the adhesive dressings in place for a few days, while some were told not to lift heavy items for a few days, but the reasons given for the counsel were not always clear and logical.

*“You know when she inserted it* (Implant)*, she told me to watch the kind of chores I will do, not to lift heavy loads with the hand that has the implant, or it might remove.”* (28-year-old; married implant user with 3 children and secondary education; Kaduna state)

### Overall quality score

**Figure 3:**
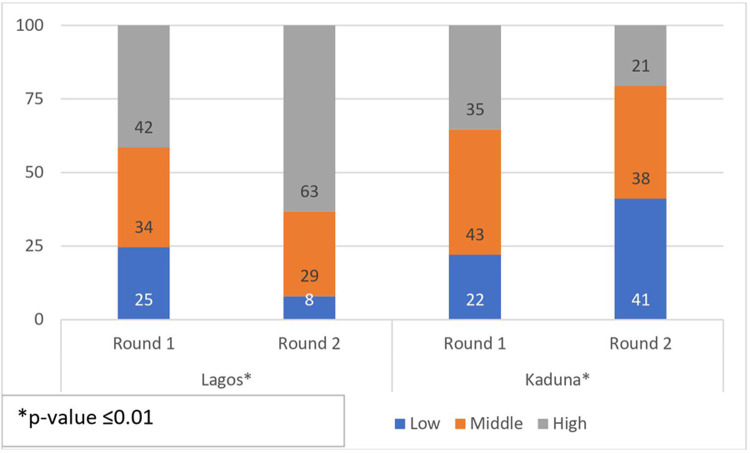
Bivariate association of quality of care by round and by state.

Figure 3 presents the results of receiving low, medium or high quality at round 1 and round 2 by state. In Lagos State, more women reported receiving high quality of care at round 2 (63%) compared to round 1 (42%), while the proportion of women who received low and medium quality care reduced. The opposite appears to have occurred in Kaduna state where more women received lower quality of care at round 2 (41%) compared to round 1 (22%). The proportion of women who received high quality care was significantly lower in round 2 (21%) compared to round 1 (35%).

## Discussion

This study explored the use of validated quality of care measures for programming and assessed how these measures worked in capturing changes in quality of care provided by CPs and PPMVs over time. In addition, we used in-depth interviews from clients to further explore clients' perception of quality of care received. While most studies to date show the effect of quality of care on contraceptive initiation, discontinuation or continuation, unique to this study is its use of validated measures to assess changes in quality of care over time within a program setting. Several studies have used secondary data sources like the DHS surveys and the PMA surveys to assess changes in the method information index (MII) over time (24, 25), none has used primary data to assess quality of care at two time points.

Our findings showed that validated quality of care measures can be used over time routinely to measure changes in quality of care. Patterns in quality of care received were not observed similarly in both Lagos and Kaduna states. The overall quality score also showed dissimilarities in the quality of care received in Lagos and Kaduna states. Women in Lagos reported receiving higher levels of quality care in round 2 compared to round 1 while in Kaduna there appeared to be a reduction in high levels of quality of care in round 2 compared to round 1. For example, in the “effective use of method selected” domain, women who were told how to manage side effects/problems increased significantly in Lagos. In Kaduna, however, this reduced significantly between round 1 and round 2. Similar to the experience observed in Kaduna among return users, a qualitative study in South Africa reported that information and family planning counseling was typically provided at contraceptive initiation but not at follow-up visits as perceived by community members (26). The decreases observed in most quality-of-care measures in Kaduna could be due to widespread insecurity associated with kidnapping, which hampered quality supervisory visits, especially to PPMVs in remote areas. During this period, providers received remote supervisory support *via* WhatsApp, suggesting perhaps that providers in Kaduna would benefit more from in-person supportive supervision visits.

In the respectful care domain, most items were nearly universally received by women in both states at both rounds and remained high. This was validated by women qualitatively interviewed as they reported they were treated well by the provider and that their questions were answered to their satisfaction. Visual privacy increased in both states at round 2. This is important as a study in Kenya showed that privacy/comfort was significantly associated with a decreased risk of discontinuation (27). Clients reported having visual privacy with an assistant present showed that quality of respectful care may not be adequate, as mentioned in this present study. When considering physical space constraints at PPMV shops and at community pharmacies, ensuring visual privacy is critical, but may require innovative approaches, when offering family planning counseling and services.

Soliciting information on clients' preferences, fertility intentions, and providing clients with information on how to use the method selected, potential side effects and how to manage them if they occur, is crucial for women to make fully informed choices around which contraceptive method they would like to use (17, 26). Our findings from both states suggest that some providers did not solicit information from clients about their reproductive intentions at round 2. For example, in both states fewer women reported being asked about their desire for another child and in Kaduna, preferred timing of next child. Because women interviewed in this study could be new contraceptive initiators or continuers, we are unable to tease apart whether CPs and PPMVs tended to ask women about fertility intentions from new users only. It is well known, however, that women's fertility intentions change and in relatively short time frames (28, 29), suggesting the need to reinforce with providers' family planning counseling approaches.

Although some clients reported that they were informed about methods that protect against STIs and components of the MII in the qualitative findings, fewer women reported receiving this information in quantitative findings from Kaduna in round 2 compared with round 1. Failure to inform women about potential side effects of contraceptive methods during the counseling encounter, and how such can be managed has been shown to be associated with higher rates of discontinuation (13). The findings from the present study suggest that providers might benefit from job aids or cue cards that remind them of aspects of quality family planning services that they can use while counseling family planning clients (30, 31).

All of the items in the continuity of contraceptive use and care domain increased significantly between rounds in Lagos, but not in Kaduna where decreases were observed in all items. This suggests that follow-up steps that are key to ensuring continuity of contraceptive use needs to be prioritized by providers offering FP counselling to clients. An earlier study in India showed that women who received information on effective use of method selected and continuity of contraceptive use and care domains were twice as likely to continue using a modern contraceptive three months after initiation (20). Using the same data, authors showed that when women received all aspects of the MII plus information about the possibility of switching methods, they were more likely to continue using a modern method three months after initiation compared to women who received the MII only (14). Continued use of contraception, irrespective of the method, should be prioritized by providers for those women who would like to prevent a pregnancy. Reasons why the items in method selection, effective use of method, and continuity of care domains reduced at round 2 in Kaduna may include that training in these areas were not complete with providers in Kaduna or that returning users did not receive full counseling as suggested by the qualitative findings.

The findings of this study are important for several reasons. First it provides some evidence that CPs and PPMVs can be trained to treat clients with respect and dignity and ensure appropriate information exchange between clients and providers. Second, findings show that quality of care can be measured in a way that can be used to track the effect of program interventions and measure quality improvement within a short period of time. It shows areas that providers need to improve their counseling skills especially in Kaduna where quality of care items reduced from round 1 to round 2. Third, these results can be used to address gaps in quality of care and improve family planning counseling and services offered by CPs and PPMVs.

Recommendations for family planning program implementation include continuous engagement of CPs and PPMVs through refresher training, supportive supervision, and provision of quick tips on key family planning counseling services that may improve information exchange between clients and providers and ensure improved quality of FP services provided. Early provision of job aids and emphasis on use may serve as reminders to providers on areas that may need to be reminded (30).

The study has several limitations. There may be slight recall issues in women's report of the quality of care received as interviews were conducted typically within two weeks of the visit to trained CPs and PPMVs, and not as they exited the respective facilities. Another limitation of the quantitative interviews is that the sample was not chosen randomly, selection of respondents was by convenience sampling of clients that visited trained CPs and PPMVs who received IntegratE's intervention package. There was no comparison group of clients who visited untrained CPs and PPMVs to assess these findings against. Finally, the results are not generalizable to all CPs and PPMVs but are useful for understanding when trained, the quality of services that these private sector providers can offer.

## Conclusion

Community Pharmacists (CP) and Patent and Proprietary Medicine Vendors (PPMVs) have been identified as key to increasing access and uptake of modern contraceptive methods. As shown by this study, these quality of care measures can be used to assess changes in care over time, which is useful for targeting programming for quality improvement. While further intervention is needed to address reduction in quality of care in a state, this study is important because it has demonstrated that providers (CPs and PPMVs) can provide high quality care and the quality of services can be improved over time. As indicated by the qualitative findings, clients who are returning family planning users also required full and complete counseling to ensure that they select the most appropriate method that meets any changes in their fertility preferences, experiences with methods, or changes in their family dynamics. Findings also suggest that the intervention needs to offer more time to train in the importance of quality of care, family planning counseling for all women irrespective of their user profile and continued supportive supervision that includes in-person interactions. Overall findings showed the need to continually build the capacity of providers to provide high quality services. There is a need for review of family planning counseling guidelines, training tools and a supportive supervision checklist to ensure quality of care standards are adhered to.

## Data Availability

The datasets presented in this study can be found in online repositories. The names of the repository/repositories and accession number(s) can be found below: https://dataverse.harvard.edu/dataverse/popcouncil.
